# The regulation mechanism of different hair types in inner Mongolia cashmere goat based on PI3K-AKT pathway and *FGF21*

**DOI:** 10.1093/jas/skac292

**Published:** 2022-09-03

**Authors:** Gao Gong, Yixing Fan, Yan Zhang, Xiaochun Yan, Wenze Li, Xiaomin Yan, Libing He, Na Wang, Oljibilig Chen, Dong He, Wei Jiang, Jinquan Li, Zhiying Wang, Qi Lv, Rui Su

**Affiliations:** College of Animal Science, Inner Mongolia Agricultural University, Hohhot 010018, China; College of Animal Science and Veterinary Medicine, Shenyang Agricultural University, Shenyang 110866, China; College of Animal Science, Inner Mongolia Agricultural University, Hohhot 010018, China; College of Animal Science, Inner Mongolia Agricultural University, Hohhot 010018, China; College of Animal Science, Inner Mongolia Agricultural University, Hohhot 010018, China; College of Animal Science, Inner Mongolia Agricultural University, Hohhot 010018, China; Inner Mongolia Jinlai Livestock Technology Co., Ltd., Hohhot 010018, China; Inner Mongolia Yiwei White Cashmere Goat Co., Ltd., Hohhot 010018, China; Inner Mongolia Yiwei White Cashmere Goat Co., Ltd., Hohhot 010018, China; Inner Mongolia Yiwei White Cashmere Goat Co., Ltd., Hohhot 010018, China; College of Animal Science, Inner Mongolia Agricultural University, Hohhot 010018, China; College of Animal Science, Inner Mongolia Agricultural University, Hohhot 010018, China; Key Laboratory of Animal Genetics, Breeding and Reproduction, Inner Mongolia Agricultural University, Hohhot 010018, China; Key Laboratory of Mutton Sheep Genetics and Breeding, Ministry of Agriculture and Rural Affairs, Hohhot,010018, China; Engineering Research Center for Goat Genetics and Breeding, Inner Mongolia Agricultural University, Hohhot 010018, China; College of Animal Science, Inner Mongolia Agricultural University, Hohhot 010018, China; College of Animal Science, Inner Mongolia Agricultural University, Hohhot 010018, China; College of Animal Science, Inner Mongolia Agricultural University, Hohhot 010018, China; Key Laboratory of Animal Genetics, Breeding and Reproduction, Inner Mongolia Agricultural University, Hohhot 010018, China; Key Laboratory of Mutton Sheep Genetics and Breeding, Ministry of Agriculture and Rural Affairs, Hohhot,010018, China; Engineering Research Center for Goat Genetics and Breeding, Inner Mongolia Agricultural University, Hohhot 010018, China

**Keywords:** different hair types, FGF21, IHC, Inner Mongolia cashmere goat, PI3K-AKT

## Abstract

There is genetic diversity of hair types in the Inner Mongolia cashmere goat population. Previous studies have found that fibroblast growth factor 21 (*FGF21*) and PI3K-AKT signal pathways may be related to different hair types in Inner Mongolia cashmere goats. Therefore, the purpose of this study was to explore the effects of the PI3K-AKT signal pathway on different hair types, the expression of mRNA and protein expression sites of *FGF21* in the hair follicles of cashmere goats with different hair types, so as to lay a foundation for understanding the molecular mechanism of different hair types and the role of skin hair follicle development. In this experiment, the skin tissues of long hair type (LHG) and short hair type (SHG) of Inner Mongolia cashmere goat were collected in three key periods of secondary hair follicle growth, namely, anagen (September), catagen (December), and telogen (March). The relative expression of *FGF21* and PI3K-AKT signal pathway candidate gene mRNA in different periods and different hair types was detected by real-time fluorescence quantitative technique (qRT-PCR), and the expression site of FGF21 protein was located by immunohistochemical technique. Through qRT-PCR, it was found that the relative expression of *FGF21*, *FGFR1*, *AKT3*, *BRCA1*, *PKN3*, *SPP1,* and *GNG4* was significantly different between LHG and SHG. The expression of *FGF21* in the skin of LHG was significantly higher than that of SHG in the three periods. Through immunohistochemical test, it was found that FGF21 protein was mainly expressed in primary hair follicle connective tissue sheath, primary hair follicle outer root sheath, secondary hair follicle outer root sheath, and sebaceous glands. It was also found that the expression of LHG skin tissue in the outer root sheath of primary hair follicles was higher than that of SHG in three periods. In summary, it is suggested that the PI3K-AKT signal pathway may play an important role in the formation of different hair types in Inner Mongolia cashmere goats.

## Teaser

The purpose of this study was to explore the role of the PI3K-AKT signal pathway in different hair types of cashmere goat hair follicles and the expression of FGF21 in different hair types of cashmere goat hair follicles. It was found that the PI3K-AKT signal pathway and FGF21 may play an important role in the formation of different hair types in Inner Mongolia cashmere goats.

## Introduction

Inner Mongolia cashmere goat is one of the most important cashmere goat breeds in China. The cashmere produced by it is of high quality and has great economic value. The skin tissue and hair follicles of cashmere goats have a very important effect on the quality and yield of cashmere (Zhang, 2011; Gong et al., 2022b). In the breeding and production of Inner Mongolia cashmere goats, it was found that cashmere goats can be further divided into long hair type cashmere goats (LHG), short hair type cashmere goats (SHG), and middle hair type cashmere goats (MHG) according to their characteristics. Through the genetic correlation analysis of cashmere and hair quality of Inner Mongolia cashmere goats, it was found that there was a negative genetic correlation between hair length and cashmere fineness, and a positive genetic correlation between hair length and cashmere length, indicating that the selection of hair type cashmere goats can indirectly improve cashmere quality (Li, 2005). To further study the classification criteria of different hair types and their effects on cashmere economic traits, the production performance and cashmere quality records of Inner Mongolia cashmere goats were analyzed by mathematical and statistical methods. It was found that hair length had significant effects on cashmere yield, cashmere length, cashmere fineness, and body weight of Inner Mongolia cashmere goats (*P* < 0.01). According to the hair length, Inner Mongolia cashmere goats can be divided into SHG (hair length ≤ 13cm), MHG (13 cm < hair length ≤ 22 cm) and LHG (hair length > 22 cm) (Na, 2016). The quantitative genetics method was used to study the different hair types in Inner Mongolia cashmere goats. It was found that the phenotypic value of cashmere yield and body weight of LHG was the highest, and the heritability and genetic correlation were the highest. The selection of LHG can accelerate the genetic progress of cashmere yield and body weight of cashmere goats and realize the indirect selection of cashmere yield and body weight (Li, 2019). To reveal the differences in skin morphology and the differentially expressed genes affecting phenotypic characteristics among different hair types, the RNA-seq technique was used to study the skin tissues of LHG and SHG for 12 consecutive months. The GO function of the different genes was mainly concentrated in intermediate filament, intermediate filament cytoskeleton and cytoskeleton, and KEGG pathway enrichment was mainly enriched in protein binding, lipid metabolism, and PI3K-AKT signaling pathway. And the key regulatory gene *FGF21* was screened (Fan, 2019; Gong, 2020; Gong et al., 2022a).

In recent years, the research on *FGF21* on skin hair follicles has become more and more abundant. Through bioinformatics methods, it was speculated that *FGF21* may promote the transformation from anagen to Catagen (Dong et al., 2013). Through the construction of the *FGF21*^−/−^ mouse model, it was found that knockout of *FGF21* could slow down hair growth, decrease hair follicle diameter and hair density, and *FGF21* would induce *AKT* expression, and then induce AKT-mediated phosphorylation of target molecules, thus affecting the complex signal network regulating hair growth (Liu et al., 2018, 2020). PI3K-AKT signaling pathway was an important signal pathway in hair follicle growth and development, which can affect the hair follicles cycle growth (Wang et al., 2021; Zhang et al., 2021), induce epidermal proliferation and epidermal progenitor cell proliferation, induce hair growth (Murayama et al., 2007), and promote hair follicle stem cell proliferation (Lv et al., 2020). PI3K-AKT signaling pathway plays an important role in new hair follicle regeneration and may provide potential therapeutic applications for hair follicle regeneration (Chen et al., 2020). The up-regulation of *VEGF* and down-regulation of *FGF5* can affect the PI3K-AKT signal pathway and improve the cashmere performance of cashmere goats with gene editing (Hu et al., 2021).

PI3K-AKT signaling pathway genes and *FGF21* play an important role in different hair types, skin, and hair follicles. Therefore, we studied their expression in different hair types of cashmere goat hair follicles and the expression site of FGF21 protein. To explore the effect of genes in the PI3K-AKT signal pathway on different hair types. To provide a scientific basis for understanding the growth and development of skin hair follicles and the breeding of cashmere goats.

## Material and Methods

### Ethics statement

In this study, skins were collected in accordance with the International Guiding Principles for Biomedical Research involving animals and approved by the Special Committee on Scientific Research and Academic Ethics of Inner Mongolia Agricultural University, responsible for the approval of Biomedical Research Ethics of Inner Mongolia Agricultural University [Approval No. (2020) 056]. No specific permissions were required for these activities, and no endangered or protected species were involved.

### Sample collection

This experiment was conducted in Inner Mongolia Jin Lai Livestock Technology Company (Hohhot, Inner Mongolia, China). According to the production performance, records and phenotypic observation data of Inner Mongolia cashmere goats, 7 LHG and 7 SHG of 2-year-old adult ewes of similar size and health were selected. Measuring the hair length near the intersection of the midline of the body and the posterior scapula. The skin tissue behind the scapula was collected in September, December, and March. One tissue was immediately put into liquid nitrogen, then stored in a cryogenic refrigerator at −80 °C for the extraction of total RNA. The other tissue was used for paraffin embedding for immunohistochemical (IHC).

### Total RNA extraction and cDNA synthesis

The skin samples were ground to powder with liquid nitrogen, then 42 samples of skin tissue total RNA were extracted with Trizol Reagent (Invitrogen) reagent. The total RNA concentration was determined by NanoDrop 2000 (Thermo), and the detection of RNA quality by 1% agarose gel electrophoresis.

cDNA was synthesized using a reverse transcription kit (PrimeScript™ RT reagent Kit with gDNA Eraser, RR047A, TAKARA). According to the instructions, 1,000 ng total RNA was taken for reverse transcription, and the cDNA reaction system was 20 μL. At the end of the reaction, the cDNA was diluted five times, and stored in the refrigerator at −20 °C.

### 2.4. PI3K-AKT signaling pathway genes analysis

Using the gene expression data (FPKM) of skin transcripts of Inner Mongolia cashmere goats with different coat types for 12 months, the gene expression information in the PI3K-AKT pathway was extracted, and the genes with low expression and no difference were filtered (Supplementary Table S1). The genes were analyzed by ggplot2 package and pheatmap package in R (Version 3.0.3), and the key genes regulating different coat types were screened.

### Real-time fluorescence quantitative PCR

Referring to the gene mRNA sequence, the fluorescence quantitative specific primers (Ye et al., 2012) of the gene were designed by using Primer-BLAST in the NCBI database. The primers were all synthesized by Sangon Biotech (Shanghai) Co., Ltd. The primer sequence is shown in Table 1.

**Table 1.** Primer sequences of genes

Gene name	NCBI reference sequence	Primer sequences		Product length (bp)	TM (°C)
*FGF21*	XM_005692688.3	F:	TGAAGCCAGGCGTCATTCAGATC	91	60
		R:	AAGTGCAGCGATCCGTACAGC		
*FGFR1*	XM_018041769.1	F:	ACAGATAACACCAAACCAAACC	214	60
		R:	TGGCATAACGGACCTTGTAG		
*AKT3*	XM_018060259.1	F:	GAACGACCAAAGCCAAACACA	213	60
		R:	AGCATCCATCTCTTCCTCTCCT		
*BRCA1*	XM_018065164.1	F:	GCCAGGGAACATCAGCCAA	156	60
		R:	TGGAAGGACGCTCAAACATCA		
*PKN3*	XM_018055908.1	F:	GCACTGAAGAAGCAGGAGGT	160	60
		R:	CAAACTCGGTCACGAAGCAG		
*SPP1*	NM_001285667.1	F:	TGAAAGCCCTGAGCAAACAGA	187	60
		R:	AGGTGGAGTGAAAACTGCGA		
*GNG4*	XM_018042602.1	F:	CACAGGGCAGAGAGTGAGTG	118	60
		R:	AGGCATACAGTGGCTTGCTT		
*GAPDH*	XM_005680968.3	F:	GCAAGTTCCACGGCACAG	118	60
		R:	TCAGCACCAGCATCACCC		
*β-actin*	XM_018039831.1	F:	GGCAGGTCATCACCATCGG	158	60
		R:	CGTGTTGGCGTAGAGGTCTTT		

Real-time quantitative PCR was performed by using a fluorescence quantitative kit (TB Green™Premix Ex Taq™ II, RR820A, TAKARA) and LightCycler ®96 Instrument (Roche). The reaction system was TB Green Premix Ex Taq II 5 μL, cDNA template 1 μL, upstream and downstream primer (10 μM) 1 μL, aseptic and enzyme-free water 3 μL. The total volume of the reaction solution is 10 μL. Three-step amplification procedure: preincubation 95 °C 30 s, 2 step amplification (95 °C 10 s, 60°C 30 s,72 °C 10 s) a total of 45 cycles, melting (95 °C 15 s, 60 °C 60 s, 97 °C 15 s), cooling (37 °C 30 s). Do three technical repeats for each sample. The expression of mRNA was calculated by 2^−−△△CT^ method (Livak and Schmittgen, 2001). The specific calculation formula is as follows: double house-keeping genes, *Cq* value calculation formula: $Cq_{house\text{\textasciitilde{}}\text{-}keeping\;genes}=\sqrt{Cq_{GAPDH}\times Cq_{\;\beta \;\text{-}actin}}$. The ratio of the difference multiple: $R=F_{LHG}/F_{SHG}$. The data were collated and summarized in excel 2019. The real-time quantitative PCR results were analyzed by SAS 9.2 ANOVA analysis of variance, SAS 9.2 CORR was used to analyze the correlation of the data and plotted by GraphPad Prism 8.3. *P* < 0.05 means the difference is significant, *P* < 0.01 means the difference is extremely significant.

### Immunohistochemical

The skin tissues of LHG and SHG of Inner Mongolia cashmere goats were selected for paraffin embedding in anagen (September), catagen (December), and telogen (March) of FGF21 protein. Use a Leica slicer (LEICA RM2235) with a thickness of 6 μm. Drying at 60 °C for 2–3 h, xylene dewaxing transparent (xylene 15 min; xylene 15 min), gradient alcohol rehydration (anhydrous ethanol 10 min; anhydrous ethanol 10 min; anhydrous ethanol 10 min, 95% alcohol 5 min, and 85% alcohol 5 min).

The experiment was carried out by BOND RX (Leica). The BOND system was programmed, the allocation capacity was set to 150 μl, the samples were set, the label is printed, and then the experiment was carried out by using BOND RX immunohistochemical instrument. The primary antibody was FGF21 rabbit anti-monoclonal antibody (ab171941, abcam), diluted to 1/100. The secondary antibody was goat anti-rabbit lgG H&L (ab205718, abcam), diluted to 1/1,000. Bond Wash Solution was used as the negative control instead of the primary antibody. BOND Program is set. Hot repair was performed with Bond ER Solution for 20 min. Quenched, incubated with 3% hydrogen peroxide at room temperature for 10 min. Wash with Bond Wash Solution for 2 min and wash four times. Seal, 5% BSA sealing solution at 37 °C for 40 min. Primary antibody incubation, incubation with FGF21 antibody at 37 °C for 120 min, cleaning. Incubate with the secondary antibody, incubate at room temperature for 30 min and wash. DAB color, DAB mixed according to the instructions, color for 10 mi, clean. Hematoxylin re-staining, stained with hematoxylin for 6 min and washed with distilled water four times. Differentiate with hydrochloric acid alcohol for 0 s and rinse with distilled water four times. Reverse blue, reverse blue with saturated lithium carbonate for 0 s, and wash with distilled water four times.

Remove the slides for dehydration and transparency, gradient alcohol dehydration (95% alcohol for 2 min; anhydrous ethanol for 2 min; anhydrous ethanol 2min), xylene transparency (xylene 5 min; xylene 5min), neutral gum seal, observed, and photographed under Nikon inverted microscope (ECLIPSETE2000-S, Nikon).

## Results

### Results of total RNA extraction from skin tissue

The quality of total RNA extraction determines the results of real-time fluorescence quantitative PCR. 1% agarose gel electrophoresis and NanoDrop2000 were used to detect the extracted skin sample RNA. Electrophoresis showed that the bands of RNA samples were clear and complete, with no degradation, with no DNA bands (Supplementary Figure S1). NanoDrop 2000 detection showed that the concentration of RNA was above 135 ng/μL, and the OD260/280 was between 1.80 and 2.01. The extracted RNA met the requirements and could be used for subsequent reverse transcription and real-time fluorescence quantitative tests.

### PI3K-AKT signaling pathway genes analysis

**Figure 1.**
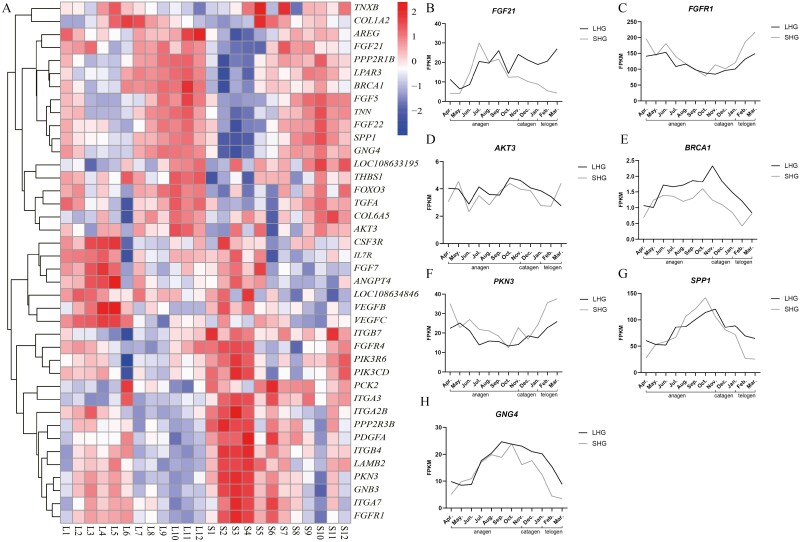
PI3K-AKT pathway gene clustering heat map and gene expression trend of core genes. (A) In the horizontal axis, L means LHG, S means SHG, 1-12 means January–December, and the vertical axis is the gene., (B–H) The horizontal axis is month, anagen (April–November), catagen (December–January), telogen (February–March), and the vertical axis is FPKM.

The PI3K-AKT signaling pathway genes were extracted from the gene expression data of different hair types in 12 months, the gene expression matrix was obtained, and the gene clustering heat map was drawn (Figure 1A). It was found that there was a difference in the expression level of these genes between the LHG and SHG, and the genes were obviously divided into two pieces. The expression level of the upper half of the genes was low from January to June and higher from July to August, and the expression of LHG was higher than that of SHG. The expression of the lower part from January to June was higher than that from July to December, and the expression of LHG was lower than that of SHG. We made a follow-up analysis of the *FGF21* and other differentially expressed genes. Draw gene expression line chart according to FPKM value.

It was found that there was no significant difference in the expression of *FGF21* between LHG and SHG during anagen, but the expression level of LHG was higher in the catagen and telogen (Figure 1B). *BRCA1* expression in LHG was higher than that in SHG (Figure 1E). There were differences in *SPP1* between LHG and SHG in anagen, catagen, and telogen (Figure 1G). *FGFR1*, *AKT3*, *PKN3,* and *GNG4* showed differences between LHG and SHG during catagen and telogen (Figure 1C, D, F, and H).

### Real-time fluorescence quantitative results

**Figure 2.**
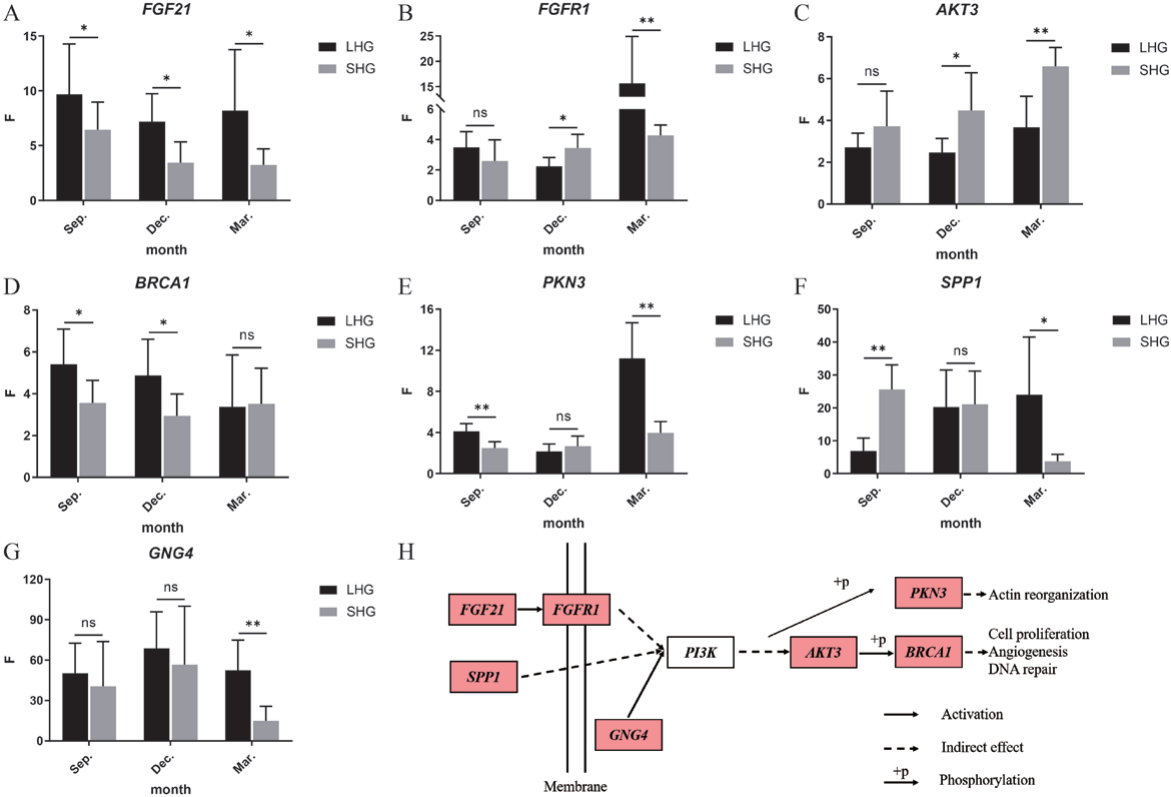
The relative expression of core genes and interactive network control chart. (A–G) The horizontal axis indicated period, anagen (September), catagen (December), telogen (March), the vertical axis was relative expression quantity *F* = 2^−△△CT^, ** indicated extremely significant difference, *indicated significant difference, ns—indicated no significant difference; (H) interactive network control chart.

The relative expression of candidate genes in the skin of LHG and SHG was determined by real-time fluorescence quantitative PCR. *GAPDH* and *β-actin* were used to calibrate the internal reference genes, and the relative expression was calculated and analyzed by analysis of variance (Figure 2, Table 2).

**Table 2.** Statistical table of relative expression of genes

Hair types	Gene name	Sep.	Dec.	Mar.
LHG	*FGF21*	11.16 ± 4.04^A^	6.88 ± 2.30^A^	8.09 ± 5.67^A^
SHG		6.06 ± 2.27^A^	3.35 ± 1.70^B^	2.71 ± 1.28^B^
*P* value		0.0427	0.0195	0.0107
LHG	*FGFR1*	3.49 ± 0.95^B^	2.24 ± 0.53^B^	15.67 ± 8.59^A^
SHG		2.74 ± 1.28^B^	3.40 ± 0.84^AB^	4.25 ± 0.64^A^
*P* value		0.1936	0.0124	0.007
LHG	*AKT3*	2.71 ± 0.63^A^	2.47 ± 0.61^A^	3.67 ± 1.38^A^
SHG		4.17 ± 1.56^B^	4.28 ± 1.67^B^	6.40 ± 0.84^A^
*P* value		0.1688	0.0174	0.0008
LHG	*BRCA1*	5.40 ± 1.56^A^	4.88 ± 1.60^A^	3.37 ± 2.29^A^
SHG		3.76 ± 1.00^A^	2.71 ± 0.98^A^	3.75 ± 1.58^A^
*P* value		0.0313	0.0259	0.9025
LHG	*PKN3*	4.13 ± 0.69^B^	2.16 ± 0.68^B^	11.22 ± 3.21^A^
SHG		2.50 ± 0.59^B^	2.41 ± 0.93^B^	3.67 ± 1.01^A^
*P* value		0.0007	0.3099	0.0002
LHG	*SPP1*	6.91 ± 3.63^B^	20.27 ± 10.43^AB^	24.01 ± 16.20^A^
SHG		25.88 ± 6.92^A^	19.33 ± 9.40^A^	4.16 ± 2.00^B^
*P* value		0.0001	0.8933	0.0101
LHG	*GNG4*	50.27 ± 20.74^A^	68.76 ± 25.13^A^	52.59 ± 20.70^A^
SHG		43.87 ± 30.77^AB^	53.55 ± 40.23^A^	15.53 ± 10.01^B^
*P* value		0.5341	0.5401	0.0017

The results showed that the relative expression of LHG in *FGF21* was significantly higher than that in SHG in three periods (*P* < 0.05). Then the analysis of variance showed that the expression level in LHG was similar in 3 months and the expression in September was significantly higher than that in December and March in SHG (*P*<0.05) (Figure 2A). The expression level of *FGFR1* in SHG was significantly higher than that in LHG in December (*P* < 0.05), and that in LHG was extremely significantly higher than that in SHG in March (*P* < 0.01), and the difference was about four times. Through the analysis of variance among the three months, it was found that the expression of LHG and SHG in March was significantly higher than that in September (Figure 2B). The expression of *AKT3* in SHG was higher than that in LHG in three periods. In December, the expression level of LHG was significantly higher than that of SHG (*P* < 0.05), and in March, the expression level of LHG was extremely significantly higher than that of SHG (*P* < 0.01). When analyzing the differences among the three periods, it was only found that the expression in March was significantly higher than that in September and December in SHG (Figure 2C). The expression of *BRCA1* in LHG was higher than that in SHG in three periods, and the expression of LHG was significantly higher than that of SHG in September and December (*P* < 0.05) (Figure 2D). The expression of *PKN3* in LHG was extremely significantly higher than that in SHG in September and March (*P* < 0.01). Then the analysis of variance between 3 months showed that the expression of 3 months in LHG and SHG was significantly higher than that in December and March (Figure 2E). The expression of *SPP1* in SHG was significantly higher than that in LHG in September (*P* < 0.05), similar in December, and significantly higher in LHG than in SHG in March (*P* < 0.05). It was found that the expression trend of LHG and SHG in the gene was significantly different, and the expression of LHG in September was significantly lower than that in December and March. The expression of SHG in March was significantly lower than that in September and December and decreased with the development of secondary hair follicles (Figure 2F). In the three periods of *GNG4*, the expression of LHG was higher than that of SHG, and only in March, the expression of LHG was significantly higher than that of SHG (*P* < 0.05) (Figure 2G).

The gene regulatory network was drawn according to the expression level of these genes and their position relationship in the PI3K-AKT signaling pathway (Supplementary Figure S2, Figure 2H). *FGF21*, *PI3K,* and *AKT3* were located at the key positions of the PI3K-AKT signaling pathway. *FGF21* gene activates *PI3K*, *PI3K* by activating membrane receptor *FGFR1* and co-activating *PI3K*, *PI3K* with *SPP1*, *GNG4*. *PKN3* was activated by a series of reactions. At the same time, *PI3K* indirectly activates *AKT3* through a series of reactions and then activates *BRCA1* by phosphorylation. And then affect the proliferation and differentiation of cells, and then regulate hair growth, and affect the phenotype of different hair types.

### Correlation analysis between gene expression and hair length

The correlation between the relative expression of core gene mRNA and hair length traits was analyzed (Table 3, Supplementary Table S2). The results showed that there was a significant positive correlation between the relative expression of *FGF21*, *FGFR1*, *BRCA1,* and *PKN3* gene and hair length. Pearson’s correlation coefficient was high, between 0.62 and 0.83. There was a significant negative correlation between the relative expression of *AKT3* and hair length. Pearson’s correlation coefficient was higher (−0.719). There was no significant correlation between the relative expression of *SPP1* and hair length.

**Table 3.** Correlation analysis between expression of genes mRNA and hair length traits

Gene name	Correlation coefficient between hair length and mRNA expression	*P value*
*FGF21*	0.71629	0.0040
*FGFR1*	0.72662	0.0032
*AKT3*	−0.71900	0.0038
*BRCA1*	0.62901	0.0160
*PKN3*	0.83336	0.0002
*SPP1*	0.11007	0.7080
*GNG4*	0.51252	0.0609

### Localization of FGF21 protein in skin tissue of cashmere goat

IHC technique was used to locate the expression site of FGF21 in both the transverse and the longitudinal sections of the skin. The nucleus of skin tissue was stained blue by hematoxylin and FGF21 protein was stained brown. Brown staining in the photo showed that FGF21 protein was expressed at this site.

**Figure 3.**
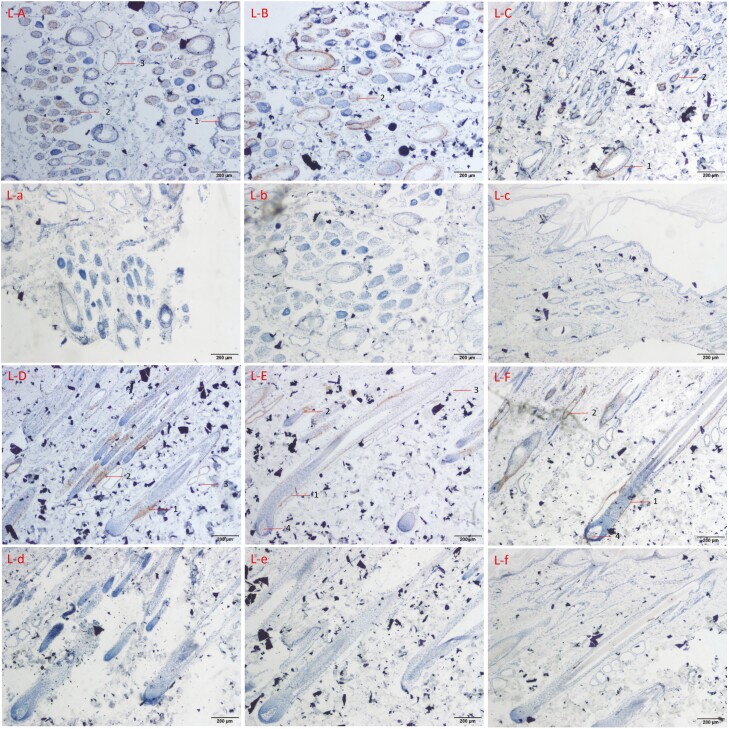
The expression position of FGF21 in three periods of LHG. L stands for LHG, S and represents SHG. A–F was used as the experimental group and a-f as the negative control group. A–C is skin transverse sections, D–F is skin longitudinal sections. A and D were skin sections in September, B and E were skin sections in December, C and F were skin sections in March. 1: outer root sheath of primary hair follicle, 2: outer root sheath of secondary hair follicle, 3: sebaceous gland, and 4: primary hair follicle hair matrix.

The IHC of FGF21 protein was carried out on the skin tissue sections of long-hair cashmere goats in September, December, and March (Figure 3). It was found that FGF21 was expressed in the skin at all three periods. It was mainly expressed in the outer root sheath of primary hair follicles, connective tissue sheath of primary hair follicles, outer root sheath of secondary hair follicles and sebaceous glands, and a little expression were also found in primary hair follicles and dermal papillae. In anagen skin tissue sections of LHG, the positive signal in the outer root sheath of secondary hair follicles was stronger than that in other periods, and it was found to be expressed in sebaceous glands. In the skin section of catagen, the positive signal of the outer root sheath in the primary hair follicle was stronger than that in other periods, and there was a small amount of positive signal in the primary hair follicle hair matrix. In telogen skin sections, the overall expression of FGF21 was small, and the secondary hair follicles were less. It could be seen that there were weak positive signals in the outer root sheath in the primary hair follicles, the hair matrix, and the outer root sheath in the secondary hair follicles. There was no positive reaction to FGF21 in the negative control group.

**Figure 4.**
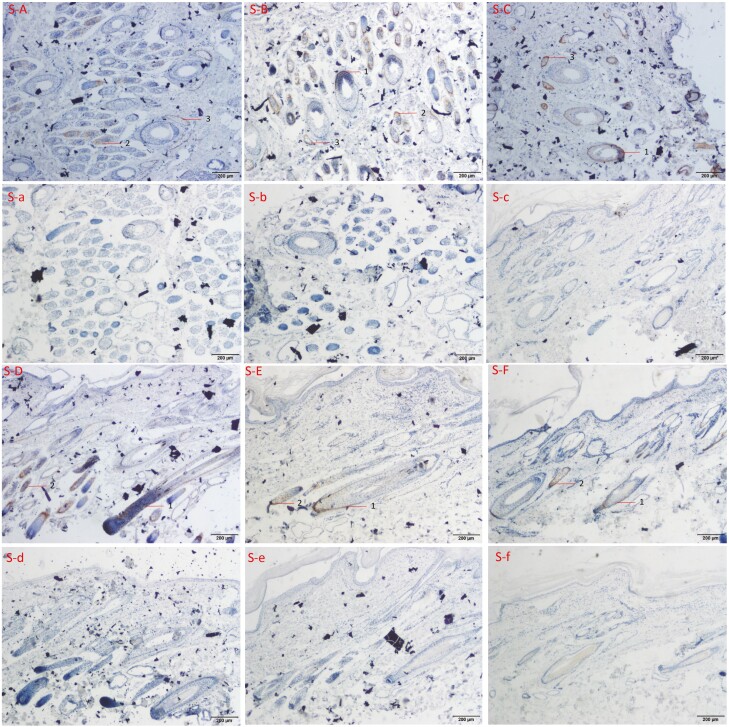
The expression position of FGF21 in three periods of SHG.

It was found that FGF21 was expressed in the skin of SHG at three periods (Figure 4). It was mainly expressed in the outer root sheath of secondary hair follicles and sebaceous glands, and a little in the outer root sheaths of primary hair follicles. In anagen skin sections of SHG, it was found that the FGF21 was expressed in the outer root sheath and sebaceous glands of secondary hair follicles, but no expression was found in primary hair follicles in tissue transverse section, but only a little expression was found in the outer root sheaths of primary hair follicles in longitudinal section. In the skin section of catagen, FGF21 was mainly expressed in the outer root sheath and sebaceous glands of secondary hair follicles, and also in the outer root sheath of primary hair follicles. In telogen skin sections, it was expressed in the root sheath and sebaceous glands in the secondary hair follicles, and there was a weak positive signal in the root sheaths in the primary hair follicles. There was no positive reaction to FGF21 in the negative control group.

Comparing the expression of FGF21 protein in the skin of LHG and SHG, it was found that there was a difference in the expression of FGF21 in the primary hair follicles of the two different hair types during the anagen, and the obvious expression of FGF21 in the primary hair follicles was observed in the skin of LHG (Figure 3 L-A, S-A). In catagen, the expression of FGF21 protein in the skin of LHG and SHG was similar, while in the primary hair follicles, the expression was different. All the outer root sheaths of the primary hair follicles were stained obviously in Figure 3 L-B, but only a few were not stained in Figure 4 S-B. The expression of FGF21 protein in the skin of LHG and SHG during the telogen was basically the same.

## Discussion

PI3K-AKT pathway is a signal pathway that controls the growth cycle of hair and can regulate the growth and development of hair follicles. At the same time, it also plays a vital role in the regeneration of new hair follicles (Chen et al., 2020). Some studies have found that 12-o-tetradecanoylphorbol-13-acetate (TPA) (Qiu et al., 2017), reactive oxygen species (ROS) (Jin et al., 2021), and ginsenoside Rb1 (Zhang et al., 2019) can activate PI3K-AKT signal pathway and promote the proliferation and hair growth of hair follicle stem cells. Activating the PI3K-AKT signal pathway can prolong the growth of hair follicles for a long time, and then achieve hair growth (Kang et al., 2020; Liu et al., 2021). In our results, we also found that genes in the PI3K-AKT pathway can affect different hair types and regulate the growth and development of hair follicles in Inner Mongolia cashmere goats.

For a long time, the role of FGFs in the development and growth regulation of hair and other skin appendages has been widely valued by researchers. Among them, *FGF5* has been proved to be an inhibitor of hair growth and applied in wool sheep (Kawano et al., 2005; Imamura, 2014; Hu et al., 2017). The study of *FGF21* in skin hair follicles is also gradually enriching. Recent studies have found that it may be a key gene that affects the hair follicle cycle and may promote the transformation of hair follicles from anagen to catagen (Dong et al., 2013). This study also confirmed that there were significant differences in the change of hair follicles cycle, and the relative expression of *FGF21* was the highest in both LHG and SHG, indicating that *FGF21* can regulate the hair follicles cycle of Inner Mongolia cashmere goats and induce them to transition from anagen to catagen. Based on the skin transcriptome data of juvenile (January) and adult (48 months) of Tan sheep, the prediction of *FGF21* may regulate the curl of hair of Tan sheep. The high expression of *FGF21* in the skin of juvenile Tan sheep was confirmed by q-PCR (Kang et al., 2013). The model of *FGF21* knockout mice was constructed by CRISPR/Cas9 system. It was found that compared with wild-type mice, *FGF21* knockout mice had lower body weight and no abnormal changes in tissues and organs. 12 days after depilation and regeneration, there was a significant difference in hair growth between the two groups, and the hair growth rate of gene knockout mice slowed down. The diameter of hair follicles, the number of hair follicles, and the density of hair decreased in gene knockout mice. *FGF21* may play an important role in the development of hair follicles (Liu et al., 2018; 2020). In Inner Mongolia cashmere goats, it was found that the expression of *FGF21* in SHG was lower than that in LHG in three periods, and the trait of hair length was also closely related to the growth rate of hair, which was similar to previous results. It is speculated that *FGF21* may affect the trait of hair length in Inner Mongolia cashmere goats. From the results of IHC, it was found that FGF21 protein was mainly expressed in the skin of cashmere goats. It was expressed in the primary hair follicle connecting tissue sheath and outer root sheath, secondary hair follicle outer root sheath, and sebaceous gland. The results of the immunohistochemical transverse section showed that no positive signal was found in the primary hair follicles in SHG in September and December, while there was a positive signal in LHG. September and December belong to anagen and catagen, and the growth of hair is relatively fast in these months. It is speculated that the differential expression of this gene in primary hair follicles may be the key factor affecting the growth of hair.

*FGFR1* has been widely studied in skin hair follicles and plays an important role in skin wound healing, hair follicle density, and hair follicles cycle. *FGFR1* can promote skin wound healing and is highly expressed in rat skin wound healing, affecting the proliferation and differentiation of epidermal keratinocytes and promoting dermal angiogenesis (Takenaka et al., 1997). *FGFR1* is the receptor on the membrane. *FGF5* can directly activate *FGFR1*, *FGFR1* together with other factors. It may bind to *Grb2* and *SOS* and activate *RRAS*. Finally, after a series of activation and phosphorylation interactions, affect cell proliferation and differentiation, and then regulate the periodic growth of hair follicles (Su et al., 2020). After knockout of the *FGF5* gene, the FGF5 protein secreted by outer root sheath cells binds to its receptor FGFR1, and the expression of *FGFR1* is down-regulated, resulting in the change of related signals in dermal papilla cells, hair fiber density, and active hair follicle density are significantly increased (Zhang et al., 2020). *FGFR1* is expressed in the papilla cells of cashmere hair in both primary and secondary hair follicles, and *FGFR1* may play a regulatory role in the growth of cashmere and hair (He et al., 2016). The expression of *FGFR1* was up-regulated during the initiation of hair follicles from 60 days to 120 days in cashmere goats (Gao et al., 2016). In this study, we also found that during telogen, the expression level of *FGFR1* is higher, which can promote the apoptosis of hair follicle structure, induce hair follicles from catagen and telogen, participate in the regulation of hair follicle cycle growth, and play a regulatory role in hair growth.

Members of the AKT gene family, including *AKT1*, *AKT2,* and *AKT3*. AKT gene family is related to the development and regeneration of skin hair follicles. The mice that knocked out the *AKT1* had growth retardation (Chen et al., 2001). Down-regulation of *AKT2* expression in *SGK3* knockout mice resulted in morphological defects of hair follicles, disorganized development of hair follicles, the disorder of cells in the outer root sheath (ORS), reduction of layers in the inner root sheath, and reduction of hair stem (McCormick et al., 2004). The hair growth of *AKT2*/*SGK3* double knockout mice had obvious defects, which accelerated the hair follicles to enter the retrogression phase, resulting in curly hair and sparse growth. It was found that both *SGK3* and *AKT2* seemed to play an important role in postpartum hair follicle morphogenesis (Mauro et al., 2009). The down-regulation of *AKT3* impaired the proliferation and migration of fibroblasts. The skin wound healing of *AKT3*^−/-^ mice was much slower than that of AKT3^+/+^ mice, indicating that AKT3 can promote wound healing (Gu et al., 2020). We found that in three periods, the expression of *AKT3* in LHG was significantly lower than that in SHG, and the expression of *AKT3* could inhibit the growth of hair length.

*BRCA1* was necessary for the formation of hair follicles and the development of hair follicle stem cells. Mice lacking *BRCA1* in the epidermis were hairless and showed a decrease in the number of degenerative hair follicles. In the process of periodic growth of hair follicles, *BRCA1* is necessary for telogen bulge stem cells. In the process of hair follicle regeneration, the loss of *BRCA1* will lead to bulge stem cell apoptosis (Sotiropoulou et al., 2013). *BRCA1* knockout mice showed moderate epidermal overproliferation and increased apoptosis (Berton et al., 2003). The down-regulation of *BRCA1* will lead to excessive proliferation of epidermis and hair removal (Deng et al., 2014). In our study, we found that the down-regulation of *BRCA1* expression in SHG showed a significant positive correlation with hair length, and this gene may promote hair growth. *SPP1* plays an important role in cell proliferation and migration, skin wound healing, cell cycle, and apoptosis (Chiarelli et al., 2019). *SPP1* can promote skin wound healing and remodeling (Liaw et al., 1998). It plays an important role in the development of skin fibrosis (Price et al., 2009; Wu et al., 2012; Ledwon et al., 2020). *SPP1* promotes hair growth by regulating the growth, proliferation, differentiation, transcriptional activation, reproduction, and tissue function of dermal papilla cells (Lin et al., 2020). In this study, we found that there was an opposite expression trend of *SPP1* in different hair types, which may indicate that different hair types of hair follicles have different rules of development.

## Conclusions

In Inner Mongolia cashmere goats, *FGF21*, *FGFR1*, *AKT3*, *BRCA1*, *PKN3*, *SPP1, GNG4,* and other genes of PI3K-AKT signal pathway genes were significant differences in the relative expression between LHG and SHG. Among them, the expression of *FGF21* in the skin of LHG was significantly higher than that of SHG in the three periods. FGF21 protein expression of LHG in the outer root sheath of primary hair follicles was higher than that of SHG in the three periods. PI3K-AKT signal pathway may regulate the formation of different hair types of Inner Mongolia cashmere goats.

## Supplementary Material

skac292_suppl_Supplementary_Figure_S1

skac292_suppl_Supplementary_Figure_S2

skac292_suppl_Supplementary_Table_S1

skac292_suppl_Supplementary_Table_S2

## Data Availability

None of the data were deposited in an official repository. The data that support the study findings are available from the authors upon request (Rui Su. Email: suruiyu@126.com).

## Abbreviations

FGF21: fibroblast growth factor 21
IHC: immunohistochemical
LHG: long hair type cashmere goat
MHG: middle hair type cashmere goats
ORS: outer root sheath
SHG: short hair type cashmere goat
