# Supplementary figures and images for: The regulation mechanism of different hair types in inner Mongolia cashmere goat based on PI3K-AKT pathway and FGF21
Source: J Anim Sci. 2022 Sep 3;100(11):skac292. doi: 10.1093/jas/skac292 (PMC9671117; doi:10.1093/jas/skac292)

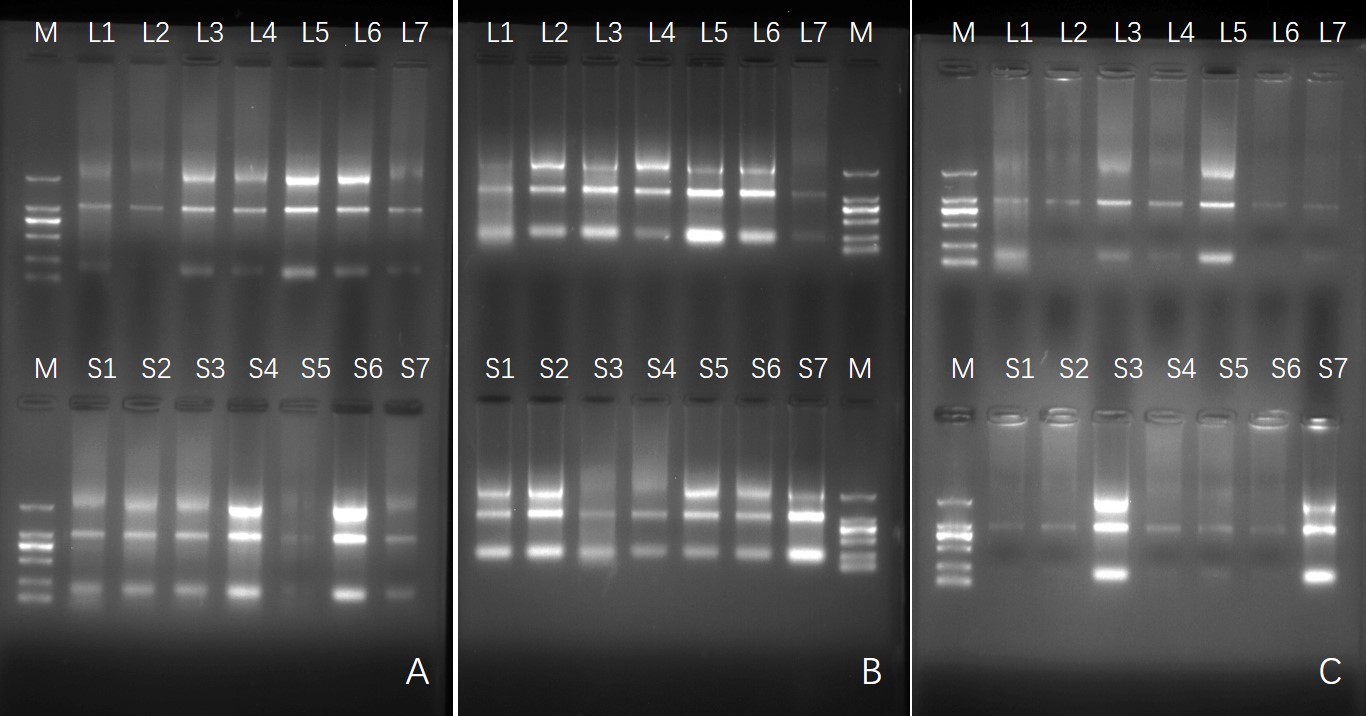

Supplement: skac292_suppl_Supplementary_Figure_S1 [file skac292_suppl_supplementary_figure_s1.jpeg]

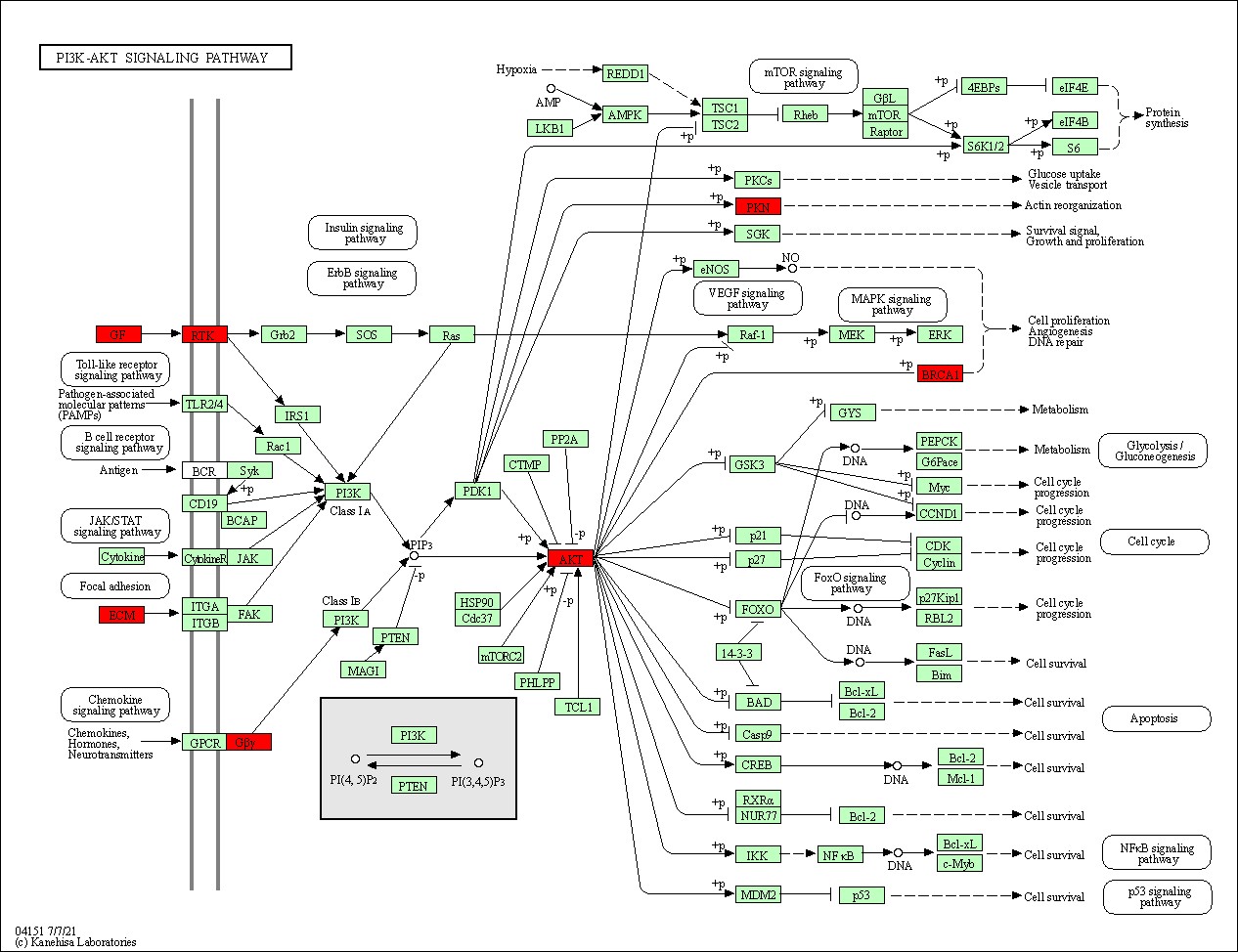

Supplement: skac292_suppl_Supplementary_Figure_S2 [file skac292_suppl_supplementary_figure_s2.jpeg]
